# Rapid Preparation of Biosorbents with High Ion Exchange Capacity from Rice Straw and Bagasse for Removal of Heavy Metals

**DOI:** 10.1155/2014/634837

**Published:** 2014-01-21

**Authors:** Supitcha Rungrodnimitchai

**Affiliations:** Department of Chemical Engineering, Faculty of Engineering, Thammasat University, Khlong Luang, Pathum Thani 12120, Thailand

## Abstract

This work describes the preparation of the cellulose phosphate with high ion exchange capacity from rice straw and bagasse for removal of heavy metals. In this study, rice straw and bagasse were modified by the reaction with phosphoric acid in the presence of urea. The introduced phosphoric group is an ion exchangeable site for heavy metal ions. The reaction by microwave heating yielded modified rice straw and modified bagasse with greater ion exchange capacities (**∼**3.62 meq/g) and shorter reaction time (1.5–5.0 min) than the phosphorylation by oil bath heating. Adsorption experiments towards Pb^2+^, Cd^2+^, and Cr^3+^ ions of the modified rice straw and the modified bagasse were performed at room temperature (heavy metal concentration 40 ppm, adsorbent 2.0 g/L). The kinetics of adsorption agreed with the pseudo-second-order model. It was shown that the modified rice straw and the modified bagasse could adsorb heavy metal ions faster than the commercial ion exchange resin (Dowax). As a result of Pb^2+^ sorption test, the modified rice straw (RH-NaOH 450W) removed Pb^2+^ much faster in the initial step and reached 92% removal after 20 min, while Dowax (commercial ion exchange resin) took 90 min for the same removal efficiency.

## 1. Introduction 

Rice straw and sugarcane bagasse are abundant agroresidues in Thailand. The sugarcane bagasse is currently used as a biofuel and in the manufacture of pulp and building materials. On the other hand, open field burning of rice straw frequently causes serious air pollution [1]. Thus a new technology for utilization of these agroresidues to a more value added material should be developed. Many researchers proposed the use of lignocellulosic waste as biosorbents for the removal of heavy metal ions in waste water (i.e., [2–6]).

The advantage of biosorbents from lignocellulosic materials is that they are biobased and biodegradable so that the use and disposal of biosorbents contribute to the reduction of theenvironmental load. On the other hand, commercial ion exchange resins are petroleum based polymers so that the cost is relatively high. Since they are nonbiodegradable, then the environmental impact for disposal is larger than the use of biosorbents.

Although biosorbents are environmental friendly and low cost, most of raw biosorbents have low metal sorption capacity because they do not contain suitable functional group for effective adsorption.

The lignocellulosic biosorbent can be modified by chemical treatment. It was reported that the adsorption capacity of wood was increased by phosphorylation of wood [7]. The enhancement of cadmium sorption capacity of juniper wood by sulfonation mainly originated from the production of sulfonic acid groups, which are binding sites for heavy metals [8]. The phosphoric acid modified rice straw showed high ability for dyes removal from aqueous solution [9]. It was found that cellulose phosphate in modified rice straw prepared by conventional heating could remove almost 100% of Cd^2+^ [10]. As shown in Table 1, rice straw and sugarcane bagasse contain approximately 30–35% and 32–43% cellulose, respectively [11].

Due to hydroxyl groups that exist in cellulose, a series of chemical reaction can easily happen. The phosphorylation of hydroxyl groups in bagasse by phosphoric acid in the presence of urea leads to the formation of cellulose phosphate:

(1) $$\begin{array}{c} {\text{H}}_{3}{\text{PO}}_{4}+\text{H–}O\text{–cellulose}\;⟶\;\text{cellulose–}O\text{–}{\text{PO}}_{3}{\text{H}}_{2} \end{array}$$

But as the low degree of substitution of cellulose phosphate is described by Inagaki et al. [12], the phosphorylation of cellulose with phosphoric acid (150°C, 8 hr) gave low degree of substitution (0.33). Microwave heating was proposed to give a rapid reaction of cellulose phosphate [13–16].

In this work, the modified rice straw by phosphorylation was compared with the modified bagasse by phosphorylation. The effect of heating methods using oil bath and microwave was discussed. Furthermore, the pretreatment of both materials using dimethyl formamide (DMF) or NaOH solution was attempted to increase the phosphorus content of the modified biosorbents. The feasibility of the modified product as cationic sorbents for removing Cd^2+^, Cr^3+^, and Pb^2+^ from aqueous solution was investigated.

## 2. Materials and Methods

### 2.1. Materials

Rice straw (*Oryza sativa*) was obtained from a local field in Ayutthaya Province, Thailand. It was washed with tap water to remove residual sugar and then dried overnight at 100°C. It was cut and ground with cooking mixer (RS). Some of rice straw was pretreated by being boiled in 16% NaOH solution for 1 hour (RS-NaOH) or immersed in DMF for 1 night (RS-DMF), washed and dried, sieved to 500 micron, and then used in chemical modification.

Sugarcane bagasse (*Saccharum* spp.) was collected from a sugarcane juice shop. Bagasse was washed with tap water to remove residual sugar and then dried overnight at 100°C. It was cut and ground with cooking mixer (Bagasse). Some of bagasse was pretreated by being boiled in 16% NaOH solution for 1 hour (Bagasse-NaOH) or immersed in DMF for 1 night (Bagasse-DMF), washed and dried, sieved to 500 micron, and then used in chemical of modification. All chemicals were reagent grade or analytical grade and used as received.

#### 2.1.1. Phosphorylation of Rice Straw and Bagasse

Rice straw or bagasse (2.00 g), urea (22.4 g, 0.37 mol), and phosphoric acid (16.8 mL, 0.29 mol) were mixed and then preheated at 80°C for 15 min in 200 mL round bottom flask. Then it was heated by oil bath at 150°C for 2 hours or by microwave irradiation (Electrolux EMM 2005) at 300 W (5.0 min), 450 W (3.0 min), 600 W (2.0 min), or 800 W (1.5 min). After cooling to room temperature, the mixture was washed with tap water to neutral pH, rinsed with acetone, and dried at 80°C. After that, the modified bagasse was immersed in 100 mL of 1.0 M HCl solution for 1 night, then washed by deionized water, and dried before analysis and sorption tests.

### 2.2. Fourier Transform Infrared (FTIR) Spectroscopy

Treated rice straw and sugarcane bagasse samples were ground and then mixed with KBr to form a disc. FTIR spectroscopy of a KBr disc containing 1% finely ground sample was performed with absorbance mode in a range of 400 to 4.000 cm^−1^.

### 2.3. Determination of Total Phosphorus

Ammonium vanadate-molybdate method was used for spectrophotometric determination of total phosphorus modified bagasse. Sample (0.02 g) and perchloric acid (2 mL) were mixed and digested at 165°C for 12 hours or until the mixtures tuned into colorless clear solution. Vanadate-molybdate acid solution (10 mL) was added to the sample (1 mL). The solution was made up to the mark (50 mL) and the absorbance was measured at 400 nm against blank sample. The total phosphorus content in samples was derived from calibration curve that was obtained using standard solution of KH_2_PO_4_ in the same spectrophotometric analytical condition.

### 2.4. Ion Exchange Capacity

Adsorbents (0.2 g) were immersed in 100 mL of 1.0 M NaCl for 12 hours. By the ion exchange reaction, parts of H^+^ in the samples were substituted by Na^+^ and give HCl solution. HCl solutions were collected and titrated with a standard NaOH solution (5 mM).

### 2.5. Sorption Experiments

Metal ion solutions for sorption experiment (40 ppm) were prepared from stock standard solution. The pH values of the solution for experiment were adjusted to 5 ± 0.1 by HNO_3_ or NaOH. Sorbents (0.2 g) were added to 100 mL of 40 ppm metal solutions. This solution was taken every fixed time for 180 minutes. Concentrations of the samples were determined using AAnalyst 800 (Perkin Elmer Instrument). The experiments were conducted in duplicate.

## 3. Results and Discussions

Reaction conditions for preparations of modified rice straw, phosphorus content, degree of substitution, and ion exchange capacity of the modified rice straw are shown in Table 2. The data of the modified bagasse are shown in Table 3.

### 3.1. Effect of Pretreatment Methods on Ion Exchange Capacity of Modified Rice Straw and Modified Bagasse

The effect of pretreatment methods on ion exchange capacity of the sample was tested by using DMF or NaOH solution. For the modified rice straw, the samples which were pretreated by DMF (RS-DMF oil 2 hr, RS-DMF 300 W, RS-DMF 450 W, RS-DMF 600 W, RS-DMF 800 W) showed slightly lower ion exchange capacity than the no pretreatment samples (RS-oil 2 hr, RS 300 W, RS 450 W, RS 600 W, and RS 800 W). However, RS-DMF oil 3 hr showed almost 2.5 times of that of RS-DMF oil 3 hr. On the other hand, for the modified bagasse, the samples which were pretreated by DMF (Bagasse-DMF oil 2 h, Bagasse-DMF 300 W, and Bagasse-DMF 450 W) showed slightly lower ion exchange capacity than the no pretreatment samples (RS-oil 2 hr, RS 300 W, and RS 450 W). But Bagasse-DMF 600 W and Bagasse-DMF 800 W showed higher ion exchange capacities than those of Bagasse 600 W and Bagasse 800 W. This may be explained by the temperature profile that DMF improved the ion exchange capacity at long reaction time or at high temperature. As seen in Tables 1 and 2, that the samples treated by NaOH solution (all RS-NaOH samples and all Bagasse-NaOH samples) gave the highest phosphorus content at every condition. It is shown that the treatment of lignocellulosic materials with NaOH solution leached out hemicellulose and lignin. It was reported that the pretreatment results in enlargement of the inner surface area of substrate particles were accomplished by partial solubilization and/or degradation of hemicellulose and lignin [17]. For this reason, the ion exchange capacities of modified samples treated by NaOH solution were much higher than those of the other kinds of modified samples. In summary, RS-NaOH oil 3 h showed the highest ion exchange capacity among modified rice straws (2.99 meq/g). RS-NaOH 450 W showed the highest ion exchange capacity among modified rice straws prepared by microwave heating (2.61 meq/g). On the other hand, Bagasse-NaOH 600 W showed the highest ion exchange capacity among modified rice straws (3.62 meq/g). It should be noted that all the modified samples treated by NaOH solution except for Bagasse-NaOH oil 2 h showed higher degree of substitution than the previously reported value (150°C, 8 hr, degree of substitution 0.33) by Inagaki et al. [12].

### 3.2. Effect of Heating Methods

From Table 1, the modified rice straw prepared by oil bath heating (RS-NaOH-oil 3 hr, 2.99 meq/g) gave lower phosphorus content (6.32%) than those of the modified rice straws prepared by microwave heating (RS-NaOH 450 W (7.07%, 2.61 meq/g), RS-NaOH 600 W (6.99%, 2.50 meq/g), and RS-NaOH 800 W (7.04%, 2.58 meq/g)). However, the ion exchange capacity of RS-NaOH-oil 3 hr was higher than the modified rice straws from microwave heating.

**Figure 1:**
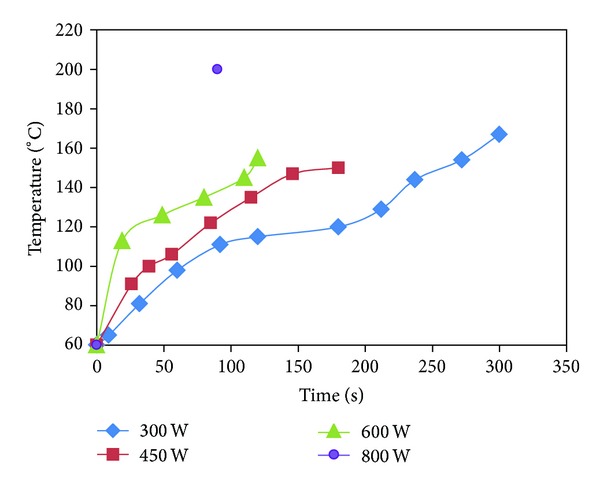
Temperature profile of phosphorylation of rice straw by microwave heating.

The modified rice straws in this work exhibited higher phosphorus contents and ion capacities than the modified rice straw (no pretreatment), which was reported by Gong and coworker (2.1% P) and also those of our previous work (2.8% P). The difference between ion capacities of the modified rice straws from conventional heating and those from microwave heating may be explained by the temperature profile of the microwave reaction (Figure 1).

In case of oil bath heating, the temperatures of the reactions were kept constantly at 150°C. But in microwave reaction at 800 W, the temperature rose up to 220°C in 90 sec. In case of 600 W (120 sec), 450 W (180 sec), and 300 W (300 sec) the temperatures at the end of reaction were about 170–180°C. All of these temperatures exceeded the optimum temperature for phosphorylation at 150°C. This may cause the side reactions and generation of by-products that contains phosphorus in microwave reaction.

**Figure 2:**
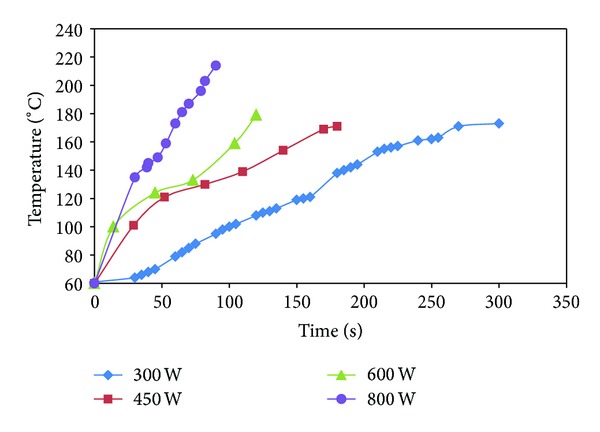
Temperature profile of phosphorylation of bagasse by microwave heating.

Temperature profile of phosphorylation of bagasse by microwave heating is shown in Figure 2. From the results in Table 2, microwave treatment was shown to be more efficient than oil bath. As described by Shibata et al. (1996), that microwave irradiation might interact directly with the reactants to cause an enhancement of the reaction rate. This is in contrast to conventional heating where heat enters the sample through its surface and is transferred towards the centre of the sample mainly by thermal conduction [18]. As a result of more effective heating, the modified bagasse prepared by microwave heating gave higher phosphorus content and faster production rate leading to very short production times. From the temperature profile of microwave reaction (Figure 2), in case of 600 W (120 sec), 450 W (180 sec) the temperature at the end of reaction was about 150–160°C which is the optimum temperature for phosphorylation, while at 800 W it rose up to 200°C in 90 sec in which this temperature exceeded the optimum temperature (150°C). Likewise, at 300 W it rose up to 150°C in 240 sec, and then the temperature increased over the optimum temperature (150°C). As seen in Table 2, the modified bagasse which has the highest phosphorus content was prepared by microwave heating at 600 W and 450 W respectively.

### 3.3. IR Spectra

**Figure 3:**
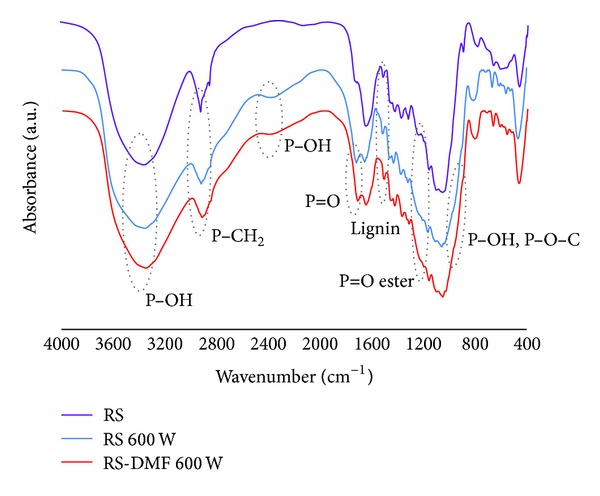
IR spectra of unmodified rice straw (RS), modified rice straw (RS 600 W), and RS-DMF 600 W.

IR spectra of unmodified rice straw (RS) and modified rice straw (untreated (RS 600 W) treated with DMF (RS-DMF 600 W)) by microwave heating at 600 W are shown in Figure 3.

The unmodified rice straw (RS) showed strong broad adsorption at 3350 cm^−1^ from vibration of –OH groups medium adsorption at 2900 cm^−1^ from –CH_2_ group, and adsorption at 1160 cm^−1^ and 1110 cm^−1^ from C–O–C bond of glycosidic or *β*-(1→4)-glycosidic bond. These absorption bands indicated the presence of cellulose unit in rice straw. Furthermore, absorption at 1510 cm^−1^ can be attributed to the vibration of aromatic units in lignin of rice straw. In the spectra of the modified rice straw (RS 600 W), a new weak absorption from P–OH bond was found at 2400 cm^−1^ and a shoulder at 2700 cm^−1^. Another absorption at 1710–1720 cm^−1^ was from the vibration of P=O bond; a shoulder at 1200–1300 cm^−1^ was from the vibration of P=O of phosphate ester. At 900–1000 cm^−1^ a shoulder from the vibration of P–OH bond and P–O–C bond was observed.

On the other hand, the spectrum of the modified rice straw treated by DMF (RS-DMF 600 W); showed almost the same absorption spectrum as that of RS 600 W. It implied that treatment by DMF led no significant change in the molecular structure of the modified rice straw.

**Figure 4:**
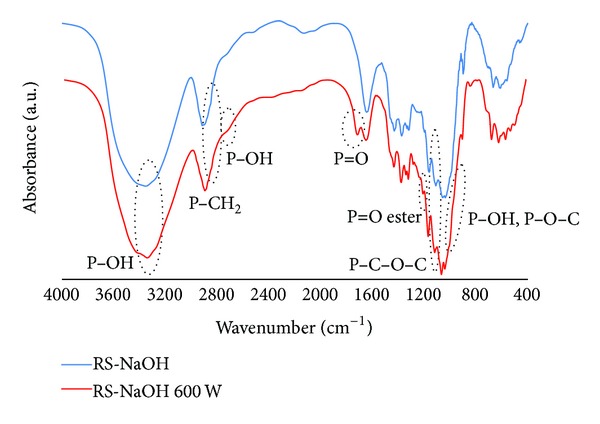
IR spectra of unmodified rice straw after treatment by NaOH solution (RS-NaOH) and modified rice straw (RS-NaOH 600 W).

IR spectra of the rice straw treated by NaOH solution (RS-NaOH), the modified rice straw (treated by NaOH solution) by 3-hour reaction in oil bath (RS-oil 3 hr), and the modified rice straw (treated by NaOH solution) by microwave heating at 600 W (RS-NaOH 600 W) are shown in Figure 4.

In Figure 4, the IR spectra of rice straw treated by NaOH before and after modification are similar to spectra of rice straw before modification (RS) and sample RS 600 W, respectively, except that the absorption at 1510 cm^−1^, which indicates the presence of lignin, disappeared. Lignin in rice straw was removed by NaOH treatment. Thus the main component of the rice straw after NaOH treatment was cellulose. The IR spectrum of RS-oil 3 hr was not different from that of RS-NaOH 600 W. The result suggests that the reaction by microwave heating gave the same product as that from the reaction in oil bath. From IR spectra, it could be concluded that the phosphoric acid group was successfully introduced into the modified rice straws.

**Figure 5:**
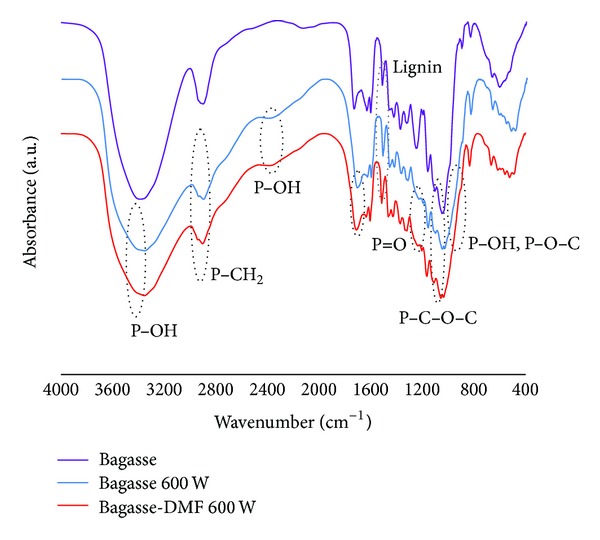
IR spectra of unmodified bagasse (Bagasse), modified bagasse (Bagasse 600 W), and Bagasse-DMF 600 W.

**Figure 6:**
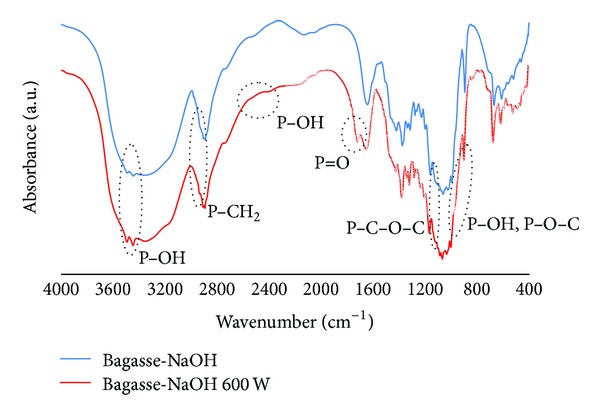
IR spectra of unmodified bagasse after treatment by NaOH solution (Bagasse-NaOH) and modified bagasse (Bagasse-NaOH 600 W).

IR spectra of the modified bagasse are shown in Figures 5 and 6. They could be explained in similar manners as those of the modified rice straw but with stronger absorption at 1710–1720 cm^−1^ from the vibration of P=O bond.

### 3.4. Kinetics Study on Adsorption of Heavy Metal Ions

**Scheme 1:**
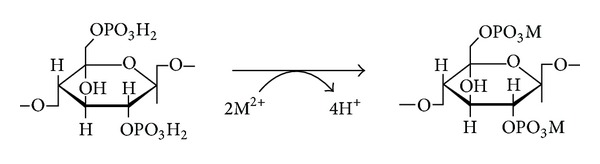
Ion exchange mechanism of cellulose phosphate.

The adsorption abilities of modified rice straws (RS-NaOH oil 3 hr and RS-NaOH 450 W) and modified bagasse (Bagasse-NaOH 600 W) were compared with the unmodified rice straw (RS), the unmodified bagasse (Bagasse), and a commercial ion exchange resin (Dowax). The ion exchange reaction is shown in Scheme 1. The ion exchange capacity of each adsorbent is listed in Table 4. The ion exchange capacities of the modified rice straw from oil bath reaction (RS-NaOH oil 3 hr), the modified rice straw from microwave reaction (RS-NaOH-450 W), the unmodified rice straw (RS), the modified bagasse from microwave reaction (Bagasse-NaOH 600 W), and Dowax were 2.99 meq/g, 2.61 meq/g, 0.12 meq/g, 3.62 meq/g, and 1.20 meq/g, respectively.

**Figure 7:**
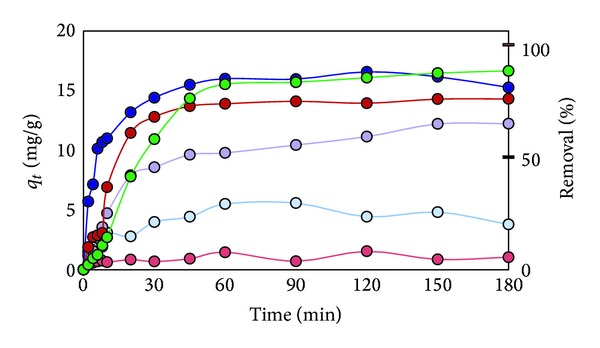
Plot of Cd^2+^ adsorption capacity at time (*q*_*t*_) and % removal versus adsorption time for effect of various sorbents (RS (light blue), RS-oil 3 hr (purple), RS-NaOH 450 W (blue), Bagasse (pink), Bagasse-NaOH 600 W (red), and Dowax (green)).

In the sorption test of 40 ppm Cd^2+^ with 2.0 g/L of the modified rice straw, the modified rice straw prepared by microwave heating (RS-NaOH-450 W) removed 85% of Cd^2+^ in 60 min, which was faster than Dowax in the first 60 min (Figure 7). It reached the highest % removal of Cd^2+^ at 120 min (88%). On the other hand, unmodified rice straw (RS) adsorbed only 29% of Cd^2+^ in 60 min. It should be noted that the modified rice straw prepared by oil bath reaction (RS-NaOH oil 3 hr), with the highest ion exchange capacity, could remove only 65% of Cd^2+^ after 180 min adsorption. Bagasse-NaOH 600 W reached its highest % removal at 180 min (76%). The unmodified rice straw (RS) and the unmodified bagasse (Bagasse) showed as low as 29.6% and 8.1% removal, respectively.

**Figure 8:**
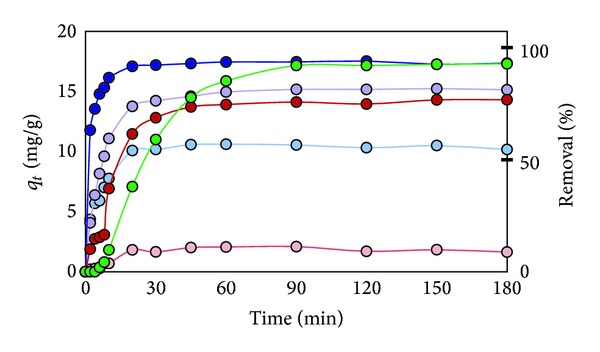
Plot of Pb^2+^ adsorption capacity at time (*q*_*t*_) and % removal versus adsorption time for effect of various sorbents (RS (light blue), RS-oil 3 hr (purple), RS-NaOH 450 W (blue), Bagasse (pink), Bagasse-NaOH 600 W (red), and Dowax (green)).

As seen in Figure 8, all of adsorbents could remove Pb^2+^ better than Cd^2+^. Especially, RS-NaOH 450 W removed Pb^2+^ much faster in the first step and reach 92% removal after 30 min, while Dowax took 90 min to give the same % removal. Apparently, the modified rice straws reached the adsorption equilibrium faster than the commercial resin. On the other hand, Bagasse-NaOH 600 W reached its highest % removal at 90 min (91%). The unmodified rice straw (RS) and the unmodified bagasse (Bagasse) showed as low as 54.59% and 8.83% removal, respectively.

**Figure 9:**
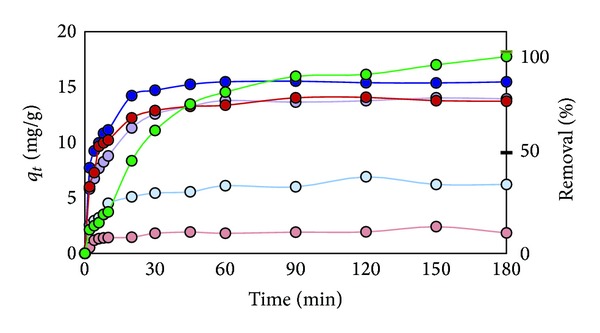
Plot of Cr^3+^ adsorption capacity at time (*q*_*t*_) and % removal versus adsorption time for effect of various sorbents (RS (light blue), RS-oil 3 hr (purple), RS-NaOH 450 W (blue), Bagasse (pink), Bagasse-NaOH 600 W (red), and Dowax (green)).

On the sorption test of Cr^3+^ ion, RS-NaOH 450 W removed 78% Cr^3+^ ion in 20 min, and the % removal attained 85% (Figure 9). RS-oil 3 hr decreased Cr^3+^ concentration to 8.5 ppm in 180 min. On the other hand, Dowax could remove 96% of ions after 180 min. On the other hand, Bagasse-NaOH 600 W reached its highest % removal at 180 min (76%). The unmodified rice straw (RS) and the unmodified bagasse (Bagasse) showed 34% and 10.2% removal, respectively.

It was reported that the adsorption kinetics of divalent ions follows pseudo-second-order kinetics [19]. The kinetics rate equation can be rewritten as follows:

(2) $$\begin{array}{c} \frac{dq_{t}}{d_{t}}=k{\left(q_{e}-q_{t}\right)}^{2}. \end{array}$$

Its integrated rate law for a pseudo-second-order reaction has a linear form of

(3) $$\begin{array}{c} \frac{t}{q_{t}}=\frac{1}{k{q_{e}}^{2}}+\frac{t}{q_{e}}. \end{array}$$

The rate constant and *q*_*e*_ can be determined experimentally by plotting of *t*/*q*_*t*_ against *t*.

**Figure 10:**
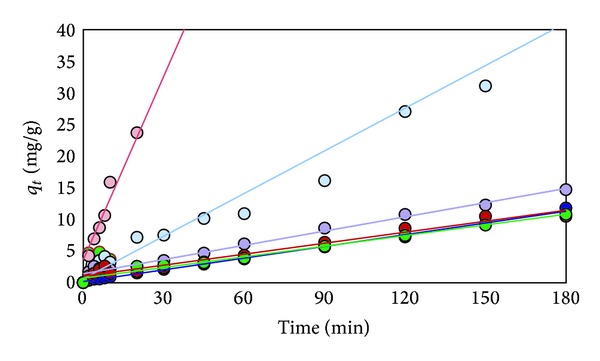
Plot of *t*/*q*_*t*_ of Cd^2+^ versus time for effect of different kinds of various adsorbents (RS (light blue), RS-oil 3 hr (purple), RS-NaOH 450 W (blue), Bagasse (pink), Bagasse-NaOH 600 W (red), and Dowax (green)).

**Figure 11:**
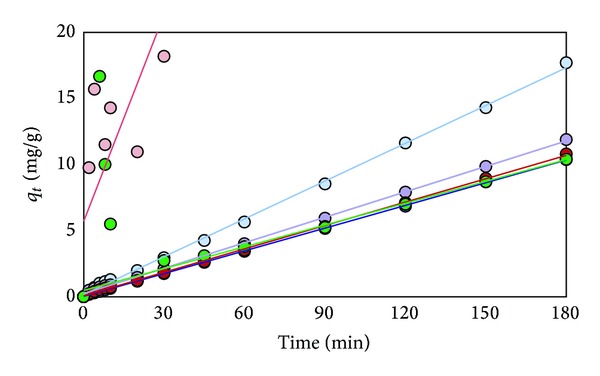
Plot of *t*/*q*_*t*_ of Pb^2+^ versus time for effect of different kinds of various adsorbents (RS (light blue), RS-oil 3 hr (purple), RS-NaOH 450 W (blue), Bagasse (pink), Bagasse-NaOH 600 W (red), and Dowax (green)).

**Figure 12:**
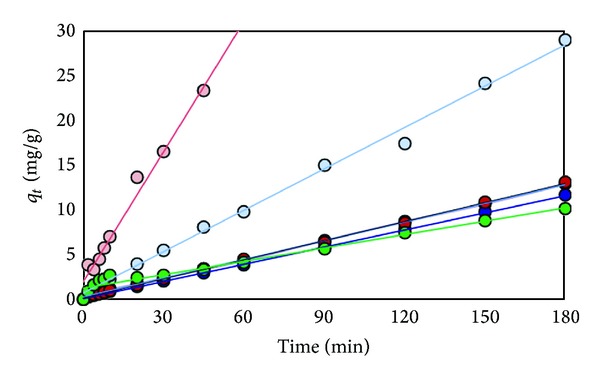
Plot of *t*/*q*_*t*_ of Cr^3+^ versus time for effect of different kinds of various adsorbents (RS (light blue), RS-oil 3 hr (purple), RS-NaOH 450 W (blue), Bagasse (pink), Bagasse-NaOH 600 W (red), and Dowax (green)).

The plots of data obtained from adsorption of Cd^2+^, Pb^2+^, and Cr^3+^are shown in Figures 10, 11, and 12, respectively. The constants derived from these plots are listed in Table 5.

Adsorption kinetics of the modified rice straws and the modified bagasse showed good fitting with the pseudo-second-order model. It was found that adsorption of the trivalent Cr^3+^ gave the best fitting results for all kinds of sorbents. On the other hand, Dowax showed a large deviation from the theoretical plot at time less than 30 min for adsorption of Cd^2+^ and Pb^2+^, so that it was fit by data of 30~180 min. For every condition, Dowax showed (ion exchange resin) the highest adsorption capacity (17.83–20.16 mg/g), and the next in the series were RS-NaOH 450 W, Bagasse-NaOH 600 W, RS-oil 3 hr, and rice straw for adsorption of Pb^2+^and Cr^3+^. On the other hand, Bagasse-NaOH 600 W showed the next highest adsorption capacity (17.27 mg/g). Although the modified bagasse (Bagasse-NaOH 600 W, 3.62 meq/g) had higher ion capacity than the modified rice straw (RS-NaOH 450 W, 2.61 meq/g), the adsorption of heavy metal ions of the modified bagasse (Bagasse-NaOH 600 W) was less effective than the modified rice straw.

It should be noted that the modified rice straw from microwave reaction (RS-NaOH 450 W) gave the largest initial rate of adsorption, which means the rapid decrease of metal concentration during adsorption. Dowax showed the smallest rate constants so that it took more time for removal of metal ions. Apparently, the modified rice straws attained adsorption equilibrium faster than the commercial resin. This result implied that the ability of adsorption of the modified bagasse was not dependent only on the ion exchange capacity.

Although RS-NaOH 450 W had lower ion exchange capacity than those of RS-oil 3 hr, it could remove more ions with faster speed. It is considered that the microwave reaction takes place at the whole porous structure of cellulose. But the oil bath reaction takes place only on the surface of the particle of cellulose. The reason that the modified rice straw showed better adsorption ability than the modified bagasse might come from its low crystallinity indices of untreated rice straw (37.7%) compared to the untreated sugarcane bagasse (44.4%), which were reported by Sakdaronnarong and Jonglertjunya [11]. So the adsorption ability of the modified rice straw from the microwave reaction may be contributed not only the ion exchange capacity but also the morphology of the material.

## 4. Conclusions

In this research, biosorbents with high ion exchanged capacities were successfully prepared from rice straw and bagasse. The treatment by NaOH solution much improved the degree of substitution and ion exchange capacities of the obtained sorbents.

The rice straw, which was pretreated by NaOH solution, gave the highest phosphorus content when it was phosphorylated by microwave at 450 W (RS-NaOH 450 W, 7.07% P, 2.60 meq/g). The reaction at 150°C for 3 hours in oil bath, gave the modified rice straw with 6.32% phosphorus and the highest ion exchange capacity for rice straw adsorbent (RS-oil 3 hr, 2.99 meq/g). On the other hand, the bagasse treated by NaOH solution gave the highest phosphorus content when it was phosphorylated by microwave at 600 W (Bagasse-NaOH 600 W, 7.76% P, 3.62 meq/g). On the adsorption experiment, both the modified rice straw and the modified bagasse could reach adsorption equilibrium faster than the commercial resin and did not show much difference of % removal from that of the commercially available ion exchange resin (Dowax). Although the modified bagasse in this work had more ion capacity than the modified rice, the modified bagasse presented a good adsorption capacity for Cd^2+^, Cr^3+^, and Pb^2+^ ions with maximum adsorption capacity as modified rice. The result suggested that the modified rice straw is a good candidate for the biodegradable ion exchange resin.

## Figures and Tables

**Table 1:** Composition of rice straw and sugarcane bagasse (Sakdaronnarong and Jonglertjunya (2012)) [11].

Composition	Rice straw	Bagasse
Cellulose	30–35%	32–43%
Hemicellulose	25–30%	19–24
Lignin	15–28%	25–32
Ashes	4–7%	2–6%

**Table 2:** Reaction conditions for preparations of modified rice straw, phosphorus content, degree of substitution, and ion exchange capacity of the modified rice straw.

Sample names	Heating methods	Conditions	% P	Degree of substitution	Ion exchange capacity (meq/g)
RS*	—	—	—	—	0.12*

RS-oil 2 hr	Oil bath	150°C 120 min	2.85	0.16	1.29
RS-oil 3 hr	Oil bath	150°C 180 min	2.11	0.12	0.99

RS 300 W	Microwave	300 W 5 min	3.03	0.17	1.30
RS 450 W	Microwave	450 W 3 min	3.75	0.22	1.73
RS 600 W	Microwave	600 W 2 min	3.66	0.21	1.64
RS 800 W	Microwave	800 W 1.5 min	3.49	0.20	1.51

RS-DMF oil 2 hr	Oil bath	150°C 120 min	2.65	0.15	1.13
RS-DMF oil 3 hr	Oil bath	150°C 180 min	5.74	0.35	2.46

RS-DMF 300 W	Microwave	300 W 5 min	3.14	0.18	1.36
RS-DMF 450 W	Microwave	450 W 3 min	3.27	0.19	1.34
RS-DMF 600 W	Microwave	600 W 2 min	3.56	0.21	1.48
RS-DMF 800 W	Microwave	800 W 1.5 min	3.14	0.18	1.24

RS-NaOH oil 2 hr	Oil bath	150°C 120 min	6.23	0.39	2.98
RS-NaOH oil 3 hr*	Oil bath	150°C 180 min	6.32	0.39	2.99*

RS-NaOH 300 W	Microwave	300 W 5 min	5.96	0.37	2.21
RS-NaOH 450 W*	Microwave	450 W 3 min	7.07	0.45	2.61*
RS-NaOH 600 W	Microwave	600 W 2 min	6.99	0.45	2.50
RS-NaOH 800 W	Microwave	800 W 1.5 min	7.04	0.45	2.58

*Samples for sorption test.

**Table 3:** Reaction conditions for preparations of modified rice straw, phosphorus content, degree of substitution, and ion exchange capacity of the modified rice straw.

Sample names	Heating methods	Conditions	% P	Degree of substitution	Ion exchange capacity (meq/g)
Bagasse*	—	—	—	—	0.11*

Bagasse oil 2 hr	Oil bath	150°C 120 min	2.90	0.16	0.86

Bagasse 300 W	Microwave	300 W 5 min	4.04	0.24	1.51
Bagasse 450 W	Microwave	450 W 3 min	4.70	0.28	2.17
Bagasse 600 W	Microwave	600 W 2 min	3.82	0.22	1.36
Bagasse 800 W	Microwave	800 W 1.5 min	2.63	0.15	0.85

Bagasse DMF oil 2 hr	Oil bath	150°C 120 min	0.95	0.05	0.25

Bagasse-DMF 300 W	Microwave	300 W 5 min	3.37	0.19	1.26
Bagasse-DMF 450 W	Microwave	450 W 3 min	4.83	0.29	1.83
Bagasse-DMF 600 W	Microwave	600 W 2 min	4.64	0.28	1.60
Bagasse-DMF 800 W	Microwave	800 W 1.5 min	4.58	0.27	1.59

Bagasse-NaOH oil 2 hr	Oil bath	150°C 120 min	2.61	0.32	1.23

Bagasse-NaOH 300 W	Microwave	300 W 5 min	5.81	0.36	2.38
Bagasse-NaOH 450 W	Microwave	450 W 3 min	7.19	0.46	3.50
Bagasse-NaOH 600 W*	Microwave	600 W 2 min	7.76	0.51*	3.62*
Bagasse-NaOH 800 W	Microwave	800 W 1.5 min	6.89	0.44	3.08

*Samples for sorption test.

**Table 4:** Ion exchange capacity of each adsorbent in the sorption test.

Adsorbents	Ion exchange capacities (meq/g)
RS	0.12
RS-NaOH oil 3 hr	2.99
RS-NaOH 450 W	2.61
Bagasse	0.11
Bagasse-NaOH 600 W	3.62
Dowax	1.20

**Table 5:** Parameters form fits to the pseudo-second-order model.

Ions	Sample	*R* ^2^	q_e_ (mg/g)	*k* (g/mg min)	Initial rate (mg/g min)
Cd^2+^	RS	0.9618	4.45	7.92*E* − 02	1.57
	RS-oil 3 hr	0.9861	13.35	3.98*E* − 03	0.71
	RS-NaOH 450 W	0.9972	16.18	2.13*E* − 02	5.58
	Baggage	0.9113	1.03	2.56*E* − 01	0.27
	Baggage-NaOH 600 W	0.9673	17.27	3.27*E* − 03	0.98
	Dowax	0.9876	17.83	4.45*E* − 03	1.41

Pb^2+^	RS	0.9988	10.50	4.94*E* − 02	5.45
	RS-oil 3 hr	0.9993	15.58	1.74*E* − 02	4.22
	RS-NaOH 450 W	0.9999	17.45	9.33*E* − 02	28.41
	Baggage	0.9528	1.92	4.76*E* − 02	0.17
	Baggage-NaOH 600 W	0.9996	16.98	3.87*E* − 02	11.16
	Dowax	0.992	18.21	6.21*E* − 03	2.06

Cr^3+^	RS	0.9957	6.50	3.43*E* − 02	1.45
	RS-oil 3 hr	0.9993	14.33	1.72*E* − 02	3.54
	RS-NaOH 450 W	0.9996	15.70	2.86*E* − 02	7.04
	Baggage	0.979	2.07	1.19*E* − 01	0.51
	Baggage-NaOH 600 W	0.9994	14.08	2.79*E* − 02	5.52
	Dowax	0.9709	20.16	1.89*E* − 03	0.77
